# A systematic review of experimental studies on *Salmonella* persistence in insects

**DOI:** 10.1038/s41538-023-00223-0

**Published:** 2023-08-28

**Authors:** Juliane Pinarelli Fazion, Filippo Marzoli, Alessandra Pezzuto, Michela Bertola, Pietro Antonelli, Beatrice Dolzan, Lisa Barco, Simone Belluco

**Affiliations:** 1 Laboratory of Safety and Quality of the Food Chain, Istituto Zooprofilattico Sperimentale Delle Venezie, Viale Fiume 78, 36100 Vicenza, Italy; 2 WOAH and Italian National Reference Laboratory for Salmonella and, Istituto Zooprofilattico Sperimentale delle Venezie, Viale dell’Università 10, 35020 Legnaro (PD), Italy; 3 Laboratory of hygiene and safety of the food chain, Istituto Zooprofilattico Sperimentale delle Venezie, Via Calvecchia 4, 30027 San Donà di Piave (VE), Italy; 4 WOAH and Italian National Reference Laboratory for Diseases at the Animal/Human Interface and Laboratory of Parasitology, Micology and Sanitary Entomology, Istituto Zooprofilattico Sperimentale delle Venezie, Viale dell’Università 10, 35020 Legnaro (PD), Italy

**Keywords:** Pathogens, Risk factors, Bacteria

## Abstract

The consumption of insects as food and feed has been recently suggested as a possible alternative to the rising global food need, thus it is crucial to monitor any potential food safety hazards in the insect supply chain. The aims of this systematic review were to collect, select, and evaluate studies investigating the persistence of *Salmonella* in insects. We searched PUBMED, EMBASE, WEB of Science Core Collection, and Food Science and Technology Abstracts. In total, 36 papers investigating the persistence of *Salmonella* in insects (both holometabolous and heterometabolous) were included after screening. Regarding complete metamorphosis insects, the longest *Salmonella* persistence was reported in *Phormia regina*, in which the pathogen persisted for 29 days at 5 °C. Similarly, *Salmonella* persisted in the feces of A*lphitobius diaperinus* for 28 days. The incomplete metamorphosis insect showing the longest *Salmonella* persistence (>10 months) was *Blatella germanica*. *Periplaneta americana* excreted *Salmonella* via feces for 44 days until all the insects were dead. The retrieved data on the persistence of *Salmonella* can be useful for further analysis by risk assessors and decision-makers involved in the safety of insect-based food, contributing to defining the sanitary requirements and risk mitigation measures along the supply chain. The review protocol is registered in PROSPERO database (CRD42022329213).

## Introduction

In recent years, the consumption of insects as food and feed has been proposed as one of the solutions to the growing demand for food worldwide, due to insects’ nutritional value, efficient conversion rate, and ecological potential^1^. Insects as food have a long and widespread history of consumption^2,3^, but in some Western countries, they are now seen as an uncommon food item. In Europe, as an example, insect-based foods (i.e., edible insects) are categorized as novel foods according to Reg. (EU) 2015/2283^4^; while in the US, insects can be used as food if they have been produced for that specific purpose following relevant rules^5^. In every case, to be defined as food, insects need to be safe with respect to foodborne hazards. Despite traditional consumption of insects not having highlighted safety concerns, as far as we know, beyond allergic reactions^1,6,7^, the scaling up of insect farms and processing plants calls for data on the behavior of foodborne pathogens in these conditions. Among the risks associated with the consumption of insects is the possible presence of foodborne pathogens, with the level of risk mainly dependent on the farming substrate^1^.

Insects have biological and ecological characteristics (i.e., ectothermy, rapid life cycle) very different from those of animals traditionally farmed for human consumption. However, as for traditionally farmed animals, some pathogens will also need to be monitored within the insect supply chain, i.e., *Salmonella*, one of the most relevant foodborne pathogens. *Salmonella* is of particular interest as it lives in the intestinal tract of humans and other animals, and possesses the ability to survive and adapt in a wide range of environments^8^. Most strains of this genus are pathogenic and are amongst the most common foodborne bacteria frequently isolated from food-producing animals that are responsible for zoonotic infections in humans and animals^9^.

A great variability in microbial loads of edible insects has been reported in the literature, mostly depending on insect species, stadium, origins (i.e., collected in nature or farmed), the killing method, and the processing of the products^10^. In the case of insect farming, the possibility of contamination by pathogenic bacteria can occur along the entire production chain, especially if basic good hygiene practices are not strictly followed. The substrate used as feed during farming ranges from feed-grade products to waste or manure so has been acknowledged as the main risk factor^1^, depending on its quality, which can be highly variable. It is noteworthy that edible insect farming and production have been developed under the impulse of sustainability, so the use of by-products is a preferable option. In this situation, pathogens like *Salmonella* can find their way to the farm and, eventually, across post-harvest processing, if they survive within insect guts or in the farming environment^11^. Thus, it is important to collect data on the persistence of *Salmonella* in farmed insects to understand and control the level of risk; this is done by identifying the conditions that favor the presence of this pathogen and by defining proper mitigation strategies to prevent contamination along the production chain.

The aims of this systematic review were to collect, select, and evaluate, from the available scientific literature, studies investigating *Salmonella* persistence in insect species.

## Results

### Study selection

**Fig. 1:**
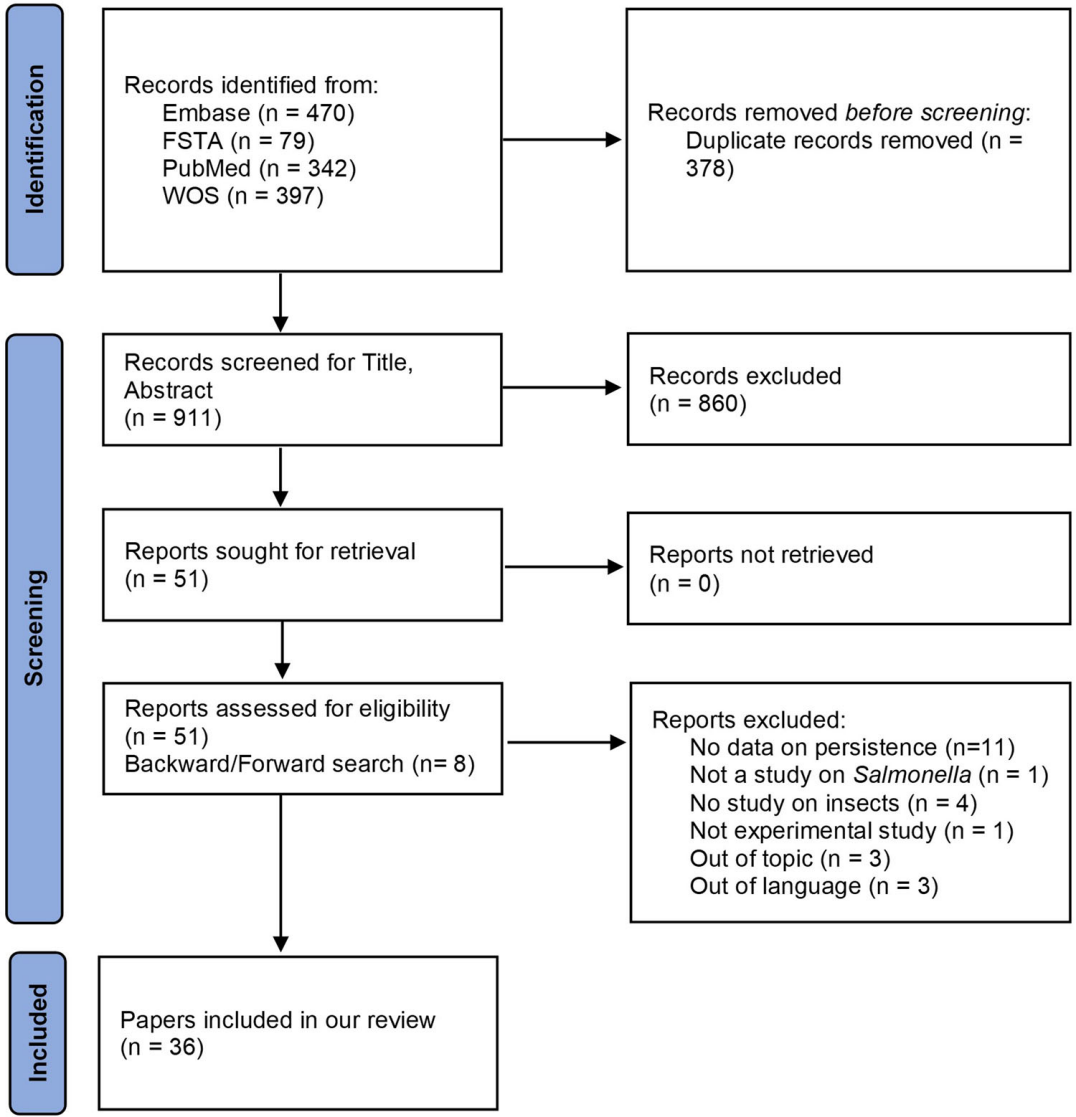
PRISMA flowchart. The PRISMA flow chart presents the results of the literature searches and the screening process.

In total, 36 papers investigating the persistence of *Salmonella* in insects were included after screening (Fig. 1). Considering that one paper investigating two insect species was considered as two different studies, in total 27 and 14 studies reported on complete metamorphosis and on incomplete metamorphosis insects, respectively.

### Study characteristics

#### Complete metamorphosis insects

Data on the general characteristics of the 27 studies carried out on complete metamorphosis insects were collected in Table 1. Concerning the geographic area where the studies were carried out, North America was the main location with 17 studies, while nine and one study were conducted in Europe and South America, respectively. Seven studies investigated the persistence of *Salmonella* in *Musca domestica* (Diptera: Muscidae), five in *Alphitobius diaperinus* (Coleoptera: Tenebrionidae), three in *Haematobia irritans* (Diptera: Muscidae), two in *Galleria mellonella* (Lepidoptera: Pyralidae), two in *Hermetia illucens* (Diptera: Stratiomyidae), two in *Tenebrio molitor* (Coleoptera: Tenebrionidae), and one study in each of the following species: *Calliphora vicina* (Diptera: Calliphoridae), *Carcinops pumilio* (Coleoptera: Histeridae), *Myzus persicae* (Rhynchota: Aphididae), *Phaenicia sericata* (Diptera: Calliphoridae), *Phormia regina* (Diptera: Calliphoridae), and *Protophormia terrae-novae* (Diptera: Calliphoridae). Two papers investigated two different insect species^12,13^. The most commonly employed techniques to analyze the persistence of *Salmonella* were based on standard microbiology (cultural and biochemical) methods. However, one study employed a biomolecular technique^11^, one study employed fluorescence-based analysis^14^, while one study did not specify the technique employed^15^.

**Table 1:** General characteristics of studies on *Salmonella* persistence in complete metamorphosis insects.

Insect order	Insect genus	Insect species	Insect life cycle stage	Temperature of insect farming (°C)	Experiment environment	Feed	*Salmonella* serotype	Contamination procedure	Country	Author
Coleoptera	*Alphitobius*	*diaperinus*	L	NR	NR	Non-medicated broiler starter crumbles	Typhimurium	CFI	US	^40^
			L, P, A	30 °C	Snap-cap tube	Wheat bran, deionized water, apple, fishmeal	Typhimurium	CFI	US	^21^
			L, A	NR	Glass containers	Rat feed and water	Paratyphi B Variant Java	DBI	NL	^41^
			L, A	NR	Petri dish	Chicken feed	Typhimurium	CFI	US	^20^
			L, A	27 °C	Plastic boxes (5.5 cm diameter x 6.0 cm high)*	Corn and soybean meal, without antibiotics	Enteritidis	CFI	BR	^22^
	*Carcinops*	*pumilio*	A	NR	Sterile petri dish	House fly eggs	Enteritidis (D + )	CFI	US	^31^
	*Tenebrio*	*molitor*	L	25.5 °C	Aluminum foil tray (19 cm × 12.5 cm × 4.5 cm, 1 L)*	Spent grain	Typhimurium DT12	CFI	DK	^24^
			L	28 °C	Aluminum tray (5 × 17 × 24 cm, 2 L)*	Wheat bran and carrot slices	Enteritidis; Typhimurium; Infantis	CFI	BE	^11^
Diptera	*Calliphora*	*vicina*	L, P, A	27 °C	Flask (1 L)*	Chick embryo	Typhimurium	CFI	US	^51^
	*Haematobia*	*irritans*	A	27 °C	Cylindrical cage (11.43 × 7.62 cm)*	Citrated bovine blood	Montevideo	CFI	US	^32^
			P, A	28 °C	Cup covered with facial tissue*	Dung	Montevideo	CFI	US	^14^
			A	NR	Square cage made of Plexiglas	Blood meal	Montevideo; Anatum; Senftenberg	CFI	US	^52^
	*Hermetia*	*illucens*	L	27 °C	Box (10 cm × 15 cm)*	Chicken feed	Typhimurium; Infantis	CFI	BE	^18^
			L	23-27-32 °C	Plastic cup (473 ml)*	Chicken manure	Enteritidis	CFI	US	^23^
	*Musca*	*domestica*	L, P	35–36 °C	Erlenmeyer flask	CSMA (wheat bran, alfalfa meal, brewers’ grain	Enteritidis; Paratyphi B; Typhi	CFI	US	^25^
			L, P	28 °C	Test tubes	NR	Schottmuller; Typhimurium; Typhi	CCS	CZ	^13^
			L, P, A	25 °C	Plastic cup (520 ml)*	Fresh poultry manure	Enteritidis	CFI	DK	^53^
			P, A	NR	Chicken laying cages*	Chicken feed and feces	Enteritidis	CFI	US	^26^
			A	22–24 °C	Jar (50 ml)	Brain hearth infusion with kanamycin and ampicillin	Typhimurium SR-11	DBI	US	^54^
			A	25–27 °C	Plastic canister and jar (946 ml)*	Manure	Typhimurium	CFI	US	^55^
			A	25 °C	Vial	5% milk / 5% sucrose solution	Typhimurium	CFI	US	^12^
	*Phaenicia*	*sericata*	A	25 °C	Vial	5% milk / 5% sucrose solution	Typhimurium	CFI	US	^12^
	*Phormia*	*regina*	P	5 °C 26 °C	Erlenmeyer flask	Larval medium (casein, yeast, powdered agar, lanolin, and Belar solution with phosphate)	Schottmuller (now Paratyphi B); Typhimurium	CFI during larval stage	US	^19^
	*Protophormia*	*terrae-novae*	L, P	28 °C	Test tubes	NR	Schottmuller; Typhimurium; Typhi	CCS	CZ	^13^
Hemiptera	*Myzus*	*persicae*	A	NR	Microcentrifuge tubes	Synthetic liquid diet, lettuce	Cubana; Enteritidis; Newport; Poona; Schwarzengrun; Baildon; Mbandaka	CFI	US	^56^
Lepidoptera	*Galleria*	*mellonella*	L	37 °C	Wood chip	No feed	Typhimurium	DI	UK	^57^
			L	37 °C	NR	NR	Typhimurium	DBI	UK	^15^

In experiment environment, the asterisk (*) means the insect farming was conducted in places different from laboratory equipment and employed a high number of insects.

*NR* not reported, *CFI* contaminated feed ingestion, *DBI* direct bacterial suspension ingestion, *DI* direct inoculation, *CCS* contact with contaminated substrate, *L* larva, *P* pupa, *A* adult, *US* United States of America, *NL* Netherlands, *BR* Brazil, *DK* Denmark, *BE* Belgium, *CZ* Czech Republic, *UK* United Kingdom.

#### Incomplete metamorphosis insects

Data on the general characteristics of the 14 studies conducted on insects with incomplete metamorphosis were collected in Table 2. The vast majority of these studies were carried out in North America (12), just one in South Asia, and one in the Middle East. Among the studies included, five were conducted on *Periplaneta Americana* (Blattodea: Blattidae), four on *Blatella germanica* (Blattodea: Blattellidae), two on *Macrosteles quadrilineatus* (Hemiptera: Cicadellidae), one on *Blaberus craniifer/Blaberus discoidalis* (Blattodea: Blaberidae), and one on *Blatta orientalis* (Blattodea: Blattidae). Moreover, one paper did not report the species of insect investigated^16^. Kopanic et al.^17^ investigated three different species of cockroaches. Also in incomplete metamorphosis insects, the most commonly employed techniques to analyze *Salmonella* persistence were based on standard microbiology methods, although one study employed fluorescence microscopy.

**Table 2:** General characteristics of studies on *Salmonella* persistence in incomplete metamorphosis insects.

Insect order	Insect genus	Insect species	Life cycle stage	Temperature of insect farming ( °C)	Experiment environment	Feed	*Salmonella* serotype	Contamination procedure	Country	Author
Blattodea	*Blaberus*	*craniifer, discoidalis*	A	NR	NR	Skim milk, yeast extract, sucrose	Typhi; Enteritidis	CFI	US	^29^
	*Blatella*	*germanica*	A	23 °C	Glass aquarium (22 L)*	Water and Purina® Dog chow® pellets	Typhimurium (83)	CFI	US	^17^
			A	NR	beaker	Sterile milk solution	Typhimurium	DBI	US	^33^
			A	22–24 °C	Glass bottle (1 L)	NR	NR	CFI	IR	^27^
			A	NR	Vessel	NR	NR	CFI	US	^34^
	*Blatta*	*orientalis*	A	23 °C	Glass aquarium (22 L)*	Water and Purina® Dog chow® pellets	Typhimurium (83)	CFI	US	^17^
	*Periplaneta*	*americana*	A	15–25 °C	Flask	Feces	Typhimurium; Montevideo	CFI	US	^30^
			A	NR	NR	5% powdered milk, yeastolate, 0.5% sucrose, 0.5% sucrose.	Typhimurium	DBI	US	^35^
			A	NR	NR	Antibiotic-milk diet; fresh cockroach feces	Typhimurium	CFI	US	^28^
			A	22–27 °C	Large glass battery jars*	NR	Typhimurium	DBI/DI/DI with latex beads	US	^36^
			A	23 °C	Glass aquarium (22 L)*	Water and Purina® Dog chow® pellets	Typhimurium (83)	CFI	US	^17^
	*Cockroach*	NR	A	NR	box*	NR	Paratyphi B Variant Java	CFI	IN	^16^
Hemiptera	*Macrosteles*	*quadrilineatus*	A	NR	Microcentrifuge tubes	Synthetic liquid diet; lettuce	Cubana; Enteritidis; Newport; Poona; Schwarzengrun; Baildon; Mbandaka	CFI	US	^56^
			A	NR	Microcentrifuge tubes	10% sucrose solution	Typhimurium	CFI	US	^58^

In the experiment environment, the asterisk (*) means the insect farming was conducted in places different from laboratory equipment and employed a high number of insects.

*NR* not reported, *CF* contaminated feed ingestion, *DBI* direct bacterial suspension ingestion, *DI* direct inoculation, *CCS* contact with the contaminated substrate, *L* larva, *P* pupa, *A* adult, *US* United States of America, *IR* Iran, *IN* India.

### Risk of bias within studies (quality evaluation)

The quality assessment of included papers is reported in Table 3. Papers reporting multiple studies did not differ from other studies with respect to our quality assessment. Control groups had been subjected to the same experimental conditions as the test groups in 17 out of 36 papers examined, and the absence of *Salmonella* in insects before artificial contamination had been ascertained in 24 papers. Almost all of the papers (34 out of 36) specified the *Salmonella* serotype used for the infection, while only two papers reported ISO methods for the qualitative and quantitative analyses of the target microorganism. Only one paper adopted farming methods similar to industrial ones. Only the paper by Wynants et al.^11^ obtained the maximum quality assessment score (5 points), while another paper achieved a score of 4^18^; all other studies were found to be deficient in at least two quality criteria.

**Table 3:** Quality assessment of included papers.

A black dot means that the paper meets the quality criteria; a white dot means that the paper is deficient for the quality criteria.

### Results of individual studies

#### Complete metamorphosis insects

Regarding complete metamorphosis insects, Table 4 shows the persistence of *Salmonella* in insects subjected to an exposure event following a period of non-exposure. The longest *Salmonella* persistence was reported in *P. regina*, in which the pathogen survived for 29 days at 5 °C. The period of persistence decreased to 5 days at 26 °C^19^. Using a high titer of contamination for insect infection (8.5 log CFU/g feed), McAllister et al.^20^ reported that *Salmonella* persisted for 28 days in the feces of *A. diaperinus*. In *A. diaperinus* infected with a lower titer of contamination (≈5 log CFU/ml), *Salmonella* was excreted through feces for up to 12 days in both larvae and adults^21^. None of these studies reported the persistence time of *Salmonella* in the substrate.

**Table 4:** *Salmonella* persistence in complete metamorphosis insects.

Insect order	Insect genus	Insect species	Insect life cycle stage	*Salmonella* serotype	Load per contaminated subject	Duration of exposure	Declaration of surface disinfection of the insect	Persistence in insects (days)	Author
Coleoptera	*Alphitobius*	*diaperinus*	L, A	Paratyphi B variant Java	4–5 log CFU/ insect	Daily for 4 weeks	No	>7	^41^
			A	Typhimurium	8.5 log CFU/g feed	24 h	Yes	>28^a^	^20^
			L	Typhimurium	8.5 log CFU/g feed	24 h	Yes	>28^a^	
			L	Typhimurium	≈5 log CFU/ml	2 h	Yes	12^a^	^21^
			A	Typhimurium	≈5 log CFU/ml	2 h	Yes	11^a^	
			L, P, A	Typhimurium	≈5 log CFU/ml	6 h	Yes	>4^b^	
			L	Typhimurium		24 h	No	7	^40^
	*Carcinops*	*pumilio*	A	Enteritidis (D+)	5.5 log CFU/egg	24 h	Yes	13; >14^a^; 4^b^	^31^
Diptera	*Haematobia*	*irritans*	A	Montevideo	1.9 log CFU/flies; 3.8 log CFU/flies; 7.3 log CFU/flies	15 min	Yes	>3	^32^
			A	Montevideo; Anatum; Senftenberg	NR	12 h	Yes	>5	^52^
			P, A	Montevideo	3 log CFU/g and 4 log CFU/g	6 days	Yes	>8^c^	^14^
	*Musca*	*domestica*	A	Typhimurium SR-11	from 5.6 log CFU /fly to 4.2 log CFU/fly	SING	Yes	>1	^54^
			A	Typhimurium	5.7 log CFU/30 g manure	12 h	No	>1	^55^
			A	Typhimurium	1.3 log CFU/insect	SING		10^a^	^12^
			A	Typhimurium	3.1 log CFU/insect	SING		>8^a^	
			L, P	Schottmuller; Typhimurium;Typhi	NR	NR	No	PDD	^13^
	*Phaenicia*	*sericata*	A	Typhimurium	2 log CFU/insect	SING		>6^a^	^12^
			A		2.9 log CFU/insect			10^a^	
			A		3.9 log CFU/insect			9^a^	
	*Phormia*	*regina*	P	Schottmuller (now Paratyphi B); Typhimurium	NR	NR	Yes	5 (at 26 °C); 29 (at 5 °C)	^19^
				Typhimurium	NR	NR	Yes	4,5 (at 26 °C); 18 (at 5 °C)	
	*Protophormia*	*terrae-novae*	L, P	Schottmuller; Typhimurium; Typhi	NR	NR	No	PDD	^13^
Hemiptera	*Myzus*	*persicae*	A	Cubana; Enteritidis; Newport; Poona; Schwarzengrund; Baildon; Mbandaka	6 log CFU/ml	24 h	No	>2 > 2^d^	^56^
Lepidoptera	*Galleria*	*mellonella*	L	Typhimurium	2–6 log/insect	IN	No	>1	^57^
			L	Typhimurium	≈0.7–2 log CFU/insect (chicken isolate SL1344)	SING	Yes	2	^15^
			L	Typhimurium	≈2 log CFU/insect (swine isolate MSG44-s01)	SING		>3	
			L	Typhimurium	≈3 log CFU/insect (laboratory isolate T4)	SING		2	

*NR* not reported, *IN* inoculation, *SING* single ingestion, *PDD* persistence during development (days not reported), *L* larva, *P* pupa, *A* adult.

^a^*Salmonella* persistence in feces.

^b^*Salmonella* persistence on insect surface.

^c^*Salmonella* persistence during metamorphosis.

^d^*Salmonella* persistence in honeydew.

Table 5 reports the persistence of *Salmonella* in complete metamorphosis insects continuously farmed on contaminated substrates. Two studies reported the persistence of *Salmonella* in *A. diaperinus* throughout the whole period of the study, showing the pathogen persisted for at least 16 days in one study^20^ and 7 days in another study^22^. Two studies reported that *Salmonella* persisted for at least 6 days in constantly exposed *H. illucens*^18,23^*. Salmonella* persistence in *T. molitor* was very variable and based on the titer of initial contamination. In particular, with substrate contaminated at 0.5, 0.8, and 2.2 log CFU/g, *Salmonella* was not detected in larvae after 1 day of exposure. However, with substrate contaminated at 5.3 log CFU/g, *Salmonella* persisted in the larvae for at least 14 days^24^. The data reported by one study are not shown in Table 5 given the impossibility for us to extract accurate data about the persistence of *Salmonella* in the different stadia of *M. domestica;*^25^ this does not affect the final persistence values, since another study reported that *Salmonella* persisted in *M. domestica* for more than 15 days^26^.

**Table 5:** *Salmonella* persistence in the farming environment of complete metamorphosis insects.

Insect order	Insect genus	Insect species	Insect life cycle stage	*Salmonella* serotype	Load per contaminated subject	Duration of exposure (days)	Declaration of surface disinfection of the insect	Persistence in the farming environment (days)	Author
Coleoptera	*Alphitobius*	*diaperinus*	L, A	Typhimurium	8.5 log CFU/g of feed	16	Yes	>16 (insect)	^20^
			L, A	Enteritidis	9 log CFU/g of feed	7	Yes	>7 (insect)	^22^
	*Tenebrio*	*molitor*	L	Typhimurium DT12	0.5 log CFU/g	14	Yes	<1 (insect); 2 (substrate)	^24^
			L		1.4 log CFU/g	14	Yes	9 (insect)	
			L		2.2 log CFU/g	14	Yes	<1 (insect)	
			L		3.8 log CFU/g	14	Yes	12 (insect)	
			L		4.1 log CFU/g	14	Yes	9 (insect)	
			L		5.3 log CFU/g	14	Yes	>14 (insect)	
			L		0.8 log CFU/g	14	No	<1 (insect); 1 (substrate)	
					1.5–5.2 log CFU/g	14	No	>14 (insect and substrate)	
			L	Enteritidis; Typhimurium; Infantis	2 log CFU/g	7	Yes and no (both techniques)	1 (insect); >7 (substrate)	^11^
			L		4 log CFU/g	7	Yes and no (both techniques)	>7 (insect and substrate)	
			L		4 log CFU/g	7	Yes	3 (insect); >7 (substrate)	
			L		7 log CFU/g	7	Yes	>7 (insect and substrate)	
Diptera	*Calliphora*	*vicina*	L, P, A	Typhimurium	NR	19	Yes	>19 (insect and substrate)	^51^
	*Hermetia*	*illucens*	L	Typhimurium; Infantis	3–7 log CFU/g of feed	6	Yes	>6 (insect and substrate)	^18^
			L	Enteritidis	6.92 log CFU/g substrate	6	No	>6 (insect)	^23^
	*Musca*	*domestica*	P, A	Enteritidis	1–4 log CFU/insect	15	No	>15 (insect)	^26^
			A	Enteritidis	9 log CFU/g substrate	8	Yes	<7 (insect); 3 (substrate)	^53^

Reported data are for *Salmonella* persistence in insects continuously farmed on a substrate contaminated with *Salmonella* on day 0.

*L* larva, *P* pupa, *A* adult, *NR* not reported.

#### Incomplete metamorphosis insects

Table 6 reports the persistence of *Salmonella* in incomplete metamorphosis insects subjected to an exposure event following a period of non-exposure. No data were found concerning incomplete metamorphosis insects continuously exposed to *Salmonella-*contaminated sources. The incomplete metamorphosis insect showing the longest *Salmonella* persistence (>10 months) was *B. germanica*^27^*. Periplaneta americana* excreted *Salmonella* via feces for 44 days until all the insects were dead^28^. Interestingly, other *Blatella* species, *B. craniifer,* and *B. discoidalis*, excreted *S*. Typhi and *S*. Enteritidis via feces for 17 and 1 day, respectively^29^. Only three authors reported the persistence of *Salmonella* in the substrate. Kopanic et al.^17^ observed that *Salmonella* can survive for more than four days in their substrate. Jung and Shaffer^30^ observed that *S*. Typhimurium and *S*. Montevideo persisted for 14 days in their substrate. According to Fathpour et al.^27^, *Salmonella* can survive for more than 45 days in their substrate, depending on whether it is dry or moist.

**Table 6:** Results of individual studies of *Salmonella* persistence in incomplete metamorphosis insects.

Insect order	Insect genus	Insect species	Insect life cycle stage	*Salmonella* serotype	Load per contaminated subject	Duration of exposure	Declaration of surface disinfection of the insect	Persistence in insects (days)	Author
Blattodea	*Blaberus*	*craniifer, discoidalis*	A	Typhi	9.3 log CFU/insect	SING	No	17^a,b^	^29^
				Enteritidis	9.5 log CFU/insect	SING	No	1^a^	
	*Blatella*	*germanica*	A	NR	NR	24 h	Yes	9	^34^
			A	Typhimurium	0–8.8 log CFU/food pellet	24 h	No	>4	^17^
			A	Typhimurium	9 different doses ranged from 2.4 log CFU/10 µl to 7.3 log CFU/10 µl	SING	No	Shortest: 3^a^; longest: 20^a^	^33^
			A	Typhimurium	9 different doses ranged from 2.4 log CFU/10 µl to 7.3 log CFU/10 µl	SING	No	Shortest: 10; longest: 29	
			A	NR	NR	SING	No	>10 months	^27^
	*Blatta*	*orientalis*	A	Typhimurium (83)	0–8.8 log CFU/food pellet	24 h	No	>4	^17^
	*NR (Cockroach)*	*NR*	A	Paratyphi B variant Java	NR	0.5 h	No	7	^16^
					NR			5^a^	
	*Periplaneta*	*americana*	A	Typhimurium; Montevideo	NR	1 h	Yes	>7	^30^
			A	Typhimurium	3.2 log CFU/insect				
					4.27 log CFU/insect	SING	No	2^a^	^35^
			A		4.4 log CFU/insect			4^a^	
			A		5.7 log CFU/insect			7^a^	
			A		6.3 log CFU/insect			16^a^	
			A	Typhimurium	4.8 log CFU/insect	SING	No	44^a,b^	^28^
			A	Typhimurium	4.8 log CFU/insect	SING	No	30^a,b,c^	
			A	Typhimurium	8 log CFU/insect	SING	Yes	6	^36^
					2 log CFU/insect	IN	Yes	4 in males	
								7 in females	
					2 log CFU/insect	SIN with latex beads	Yes	9 in males	
			A	Typhimurium (83)	0–8.8 log CFU/food pellet	24 h	No	>4	^17^
Hemiptera	*Macrosteles*	*quadrilineatus*	A	Cubana; Enteritidis; Newport; Poona; Schwarzengrund; Baildon; Mbandaka	5.7 log CFU/insect	24 h	No	>2	^56^
								>2^d^	
			A	Typhimurium	NR	12 h	No	>2	^58^

*NR* not reported, *IN* inoculation, *SING* single ingestion, *A* adult.

^a^*Salmonella* persistence in feces.

^b^until insect death.

^c^from day 10 fed with cockroach feces.

^d^*Salmonella* persistence in honeydew.

### Synthesis of results

**Fig. 2:**
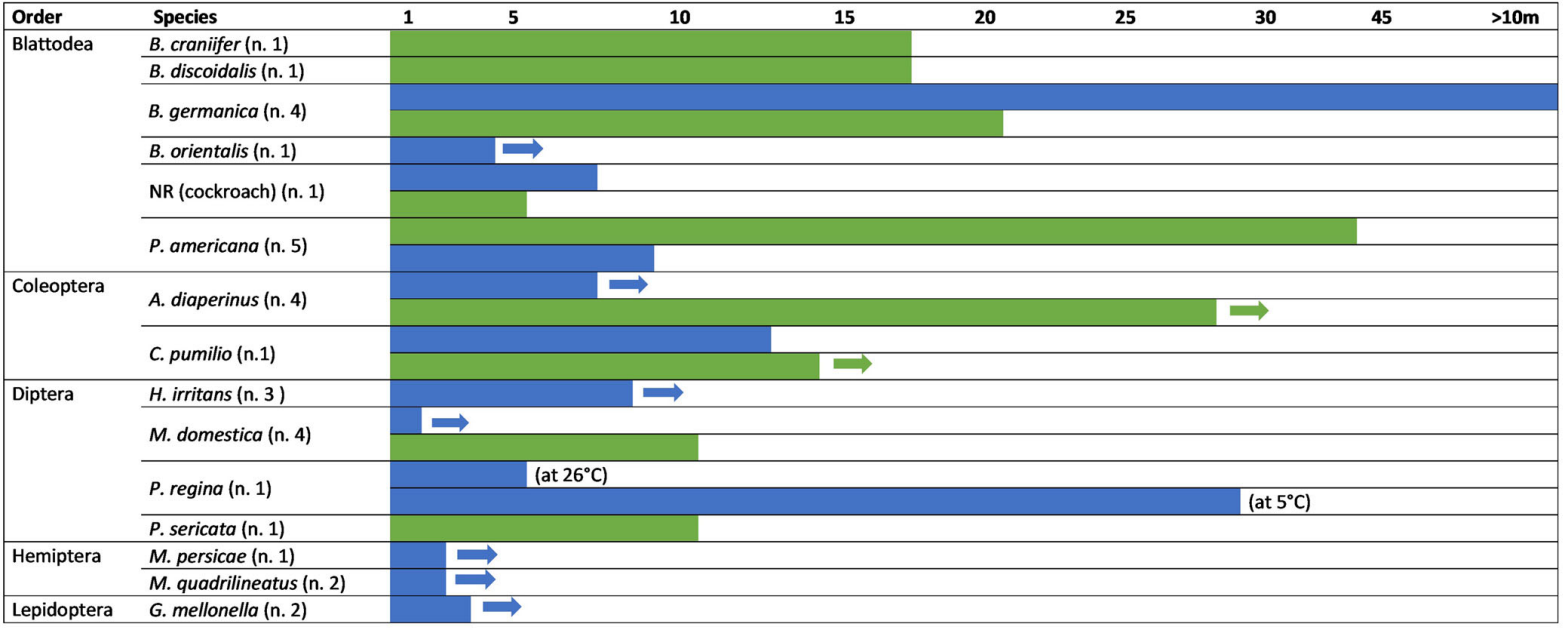
Maximal persistence (in days) of *Salmonella* in complete and incomplete metamorphosis insects. Blue bars indicate persistence in insects; green bars indicate persistence in feces. Arrows indicate that *Salmonella* persisted throughout the whole period of the study. >10 m: persistence for more than 10 months; n.: number of studies in each species.

Considering both complete and incomplete metamorphosis insects, the longest *Salmonella* persistence in an insect was recorded in *B. germanica* for a period of 10 months^27^ (Fig. 2). On the other hand, the longest duration of *Salmonella* excretion via feces was registered in *P. americana*, since the pathogen was detected for 44 days^28^. One study reported that *Salmonella* persisted on *C. pumilio* surface for four days^31^. It is important to note that the longest persistence of *Salmonella* in insects was shown in incomplete metamorphosis insects. *A. diaperinus* was the complete metamorphosis insect showing the longest duration of *Salmonella* excretion via feces^20^, while in an insect, the longest *Salmonella* persistence was in *C. pumilio*^31^.

**Fig. 3:**
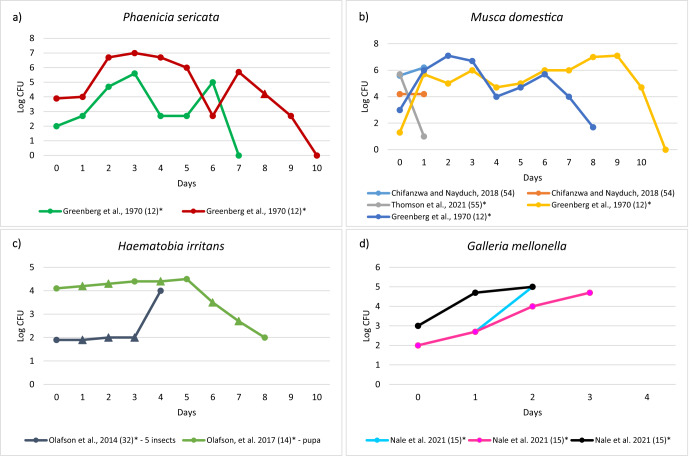
Salmonella counts over time in complete metamorphosis insects. The figure shows the trends of Salmonella counts over time (days) for the following insect species: **a** *Phenicia sericata*, **b** *Musca domestica*, **c** *Haematobia irritans*, **d** *Galleria mellonella*. (*) Data extracted manually from figures; Δ: values manually added for technical reasons with the aim of not affecting the trend of the persistence curves.

Figure 3 shows the six studies that reported the counts of *Salmonella* over time in complete metamorphosis insects. The highest counts of *Salmonella* were reported in *M. domestica* and, interestingly, this level was reached nine days after contamination^12^. *Salmonella* counts in *G. mellonella* were monitored for no more than three days and an increasing trend was observed in all cases^15^. Two studies investigated *Salmonella* counts in *H. irritans*, and both showed an increase in *Salmonella* counts in the first four days, while one study reported a decrease from day five^14,32^.

**Fig. 4:**
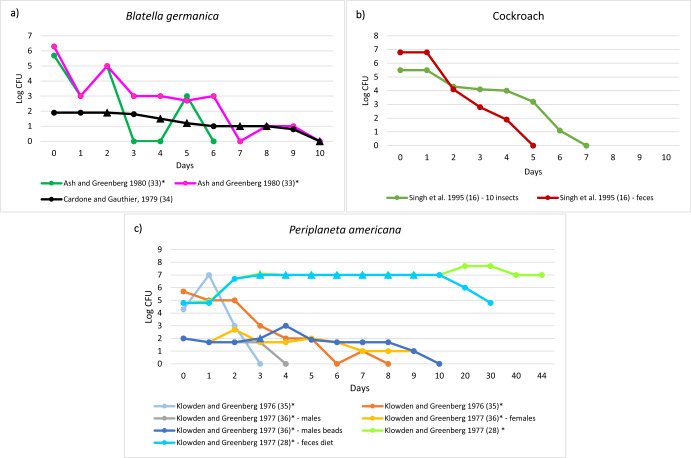
Salmonella counts over time in incomplete metamorphosis insects. The figure shows the trends of Salmonella counts over time (days) for the following insect species: **a** *Blatella germanica*, **b** Cockroach (species not reported), **c** *Periplaneta americana*; (*) Data extracted manually from figures; Δ: values manually added for technical reasons with the aim of not affecting the trend of the persistence curves.

*Salmonella* counts over time in incomplete metamorphosis insects were reported by six studies (Fig. 4). Only one study, carried out in *P. americana*, showed a marked increase of *Salmonella* counts during the insect life cycle and a long persistence until insect death^28^. All the other studies showed declines in *Salmonella* counts within 10 days^16,33–36^.

## Discussion

*Salmonella* was the second most common zoonotic agent in the European Union (EU) in 2021, so for this reason, it is important to understand this pathogen’s role in novel foods, such as insect-based food. Many animals can harbor *Salmonella*, often without any symptoms, and excrete the bacteria into the environment with the potential transmission to other animals, crops, and water reservoirs. As a result, contaminated substrate, insufficient hygienic measures, or lack of measures for preventing the entrance of undesired pests can all cause the introduction of *Salmonella* into insect production facilities. Therefore, safety hazards have to be monitored during the farming and processing of insects to ensure a safe final product^37^. Data about the ability of *Salmonella* to persist in the farming environment or inside insects could be pivotal in the risk assessment process for insect-based foods.

Gathering information about this risk is very important, as there is a need to explore substrates for insect farming that are not yet allowed but that can further boost the contribution of the sector to a circular economy (i.e., former foodstuffs containing meat, slaughter waste, etc.). This was also identified as a research priority by the International Platform of Insects for Food and Feed (IPIFF)^38^. These kinds of substrates could present a serious risk of insect contamination/infection with *Salmonella*.

Even if it has been observed that edible insects and derived products pose a low risk regarding *Salmonella*^10,39^, we can speculate that such risk has not been assessed in a proper way due to the lack of specific studies and due to the fact that such risk increases with farm dimensions and, thus, can be expected to be more relevant in the future. Few studies have been carried out on insect species that have potential as edible food. Only four studies were conducted on *A. diaperinus* subjected to an exposure event following a period of non-exposure^20,21,40,41^, and only two studies were conducted on each of *A. diaperinus*^20,22^ and *T. molitor*^11,24^ continuously farmed on a contaminated substrate. No studies were conducted on the persistence of *Salmonella* in other important species relevant to food production (i.e., *Acheta domesticus* and *Locusta migratoria*).

Considering the risks associated with the farming environment, the data reported in Table 5 could represent real conditions of *Salmonella* persistence, since the insects are continuously exposed to the same substrate during the whole cycle of farming. It is worth noting that some insect species have been observed to reduce or even eliminate some pathogens in their substrate, probably due to the efficient defense mechanisms (i.e., antimicrobial peptides)^42–44^. For example, *H. illucens* was able to reduce, in their feces, *Salmonella* from different animal species and in resultant dog feed prepared from the insects^23,45–47^. However, in general, it has been shown that humid farming conditions for *T. molitor* and the addition of wet substrate as a water source could facilitate *Salmonella* growth and persistence^24^. Interestingly, some studies reported that *T. molitor* larvae do not retain *Salmonella* when present at low levels in the substrate, likely due to competitive exclusion by the endogenous larval microbiota and/or antimicrobial peptide production by the larvae^11,24^.

Even if the complete metamorphosis insects face an extensive change in microbiota between the larval and adult stages due to the gut replacement during pupation^48,49^, some studies reported that *Salmonella* persisted during the metamorphosis from larva to adult. In particular, this phenomenon was observed in *A. diaperinus*^21^*, H. irritans*^14^*, M. domestica*, and *P. terrae-novae*^13^ (Table 4).

The risk assessment related to the presence and persistence of *Salmonella* in insect farming is also of relevance for the processing of raw insects since these food production activities are generally a critical line of defense against potential hazards. Indeed, it has been shown that in raw insects contaminated with *Salmonella*, mild treatments, such as solar-drying and oven-drying (60 °C for 2–3 days), were not effective for *Salmonella* elimination^50^.

The greatest limitation is that few studies were conducted on insect species currently relevant for food production. For example, no studies were found on *Salmonella* persistence in *A. domesticus* or *L. migratoria*.

Another important limitation is related to the high heterogeneity of the design of the included studies (duration of exposure, contamination procedure, experiment environment, etc.), which did not allow statistical synthesis of study results.

We considered only scientific literature published in six languages, but due to the long tradition of edible insect consumption in eastern Asia, it is possible that relevant studies, not published in these languages, were missed. Another limitation is due to the exclusion of gray literature. For quality purposes, we decided to base our investigation on peer-reviewed papers.

This review on the persistence of *Salmonella* collected data useful for risk assessors and risk managers involved in the study and decision-making processes regarding the safety of insect-based foods. In particular, these data can contribute to defining the hygienic-sanitary requirements and risk mitigation measures along the supply chain. To assess the risk in a complete way, data on the prevalence of *Salmonella* in the investigated species or derived products are needed. Therefore, future research should focus on this, with a particular focus on insect species with potential as food or feed. To guarantee data uniformity and allow comparison of *Salmonella* persistence in insects, we recommend the definition of a species-specific reference study protocols.

## Methods

### Review question, eligibility criteria, information sources, and search strategies

The review question was: “How long can *Salmonella* survive in farmed insects?” Key elements were identified as follows: Population: insects; Intervention: contamination with *Salmonella*, Outcome: *Salmonella* persistence (days). We considered all studies published in peer-reviewed journals in English, French, Italian, Portuguese, German, and Spanish languages. No time limits were imposed. We searched PUBMED, EMBASE, WEB of Science Core Collection, and Food Science and Technology Abstracts (FSTA). The last date searched was March 2nd, 2022. The keywords related to insects were based on the list of insect species that, at the European level, were considered highly likely to be used as food and feed^1^. Specifically, we used as keywords the order, genus, and popular name of the listed insect species. Details about the search strategy are available in Supplementary Table 1.

Several criteria were used to select eligible studies: (1) reported data had to belong to primary research; (2) the study had to involve *Salmonella*; (3) the study had to report data from experimental studies (experimental contamination with *Salmonella*); (4) the study had to deal with insects; (5) the study had to report results about *Salmonella* persistence. To increase the sensitivity of the search process, we used the final list of included papers to carry out a backward and forward reference search in order to identify potential missing evidence. The review protocol is registered in PROSPERO database (CRD42022329213).

### Selection and data collection processes

The screening process was carried out using the Parsifal online software (https://parsif.al/). Six reviewers (F.M., A.P., M.B., P.A., B.D., S.B.) categorized all studies obtained via the initial literature search based on title and abstract. In the case of a poorly explicative abstract or in the case of doubt about the available data, the study was included and evaluated at the full-text level. Each record was coded twice, i.e., separately by two reviewers, and a third reviewer solved conflicts. All studies were coded according to the previously chosen eligibility criteria.

After full-text retrieval, six reviewers (J.P.F., F.M., A.P., M.B., P.A., B.D.) extracted data from the included studies. Data were extracted from text, tables, or figures and were entered into pre-defined tabular forms. Extracted data were controlled by two reviewers independently cross-checking the extracted data with the original data in the studies.

### Data items

We defined “study” as an investigation reporting data for *Salmonella* persistence on a single insect species (i.e., one paper investigating two insect species was considered as two different studies). General data related to the included studies were listed in tables reporting the following information: (*i)* insect order; *(ii)* insect genus; (*iii)* insect species; (*iv)* insect life cycle stage; (*v)* temperature of insect farming; *vi)* experiment environment; *(vii)* feed; *(viii) Salmonella* serotype; (*xi)* contamination procedure; *x)* country where the study was performed; *(xi)* author.

### Synthesis methods

The persistence (in days) of *Salmonella* in insect species was reported in tables that included additional data useful to highlight the heterogeneity of the included studies: *(i)* insect order*; (ii)* insect genus; *(iii)* insect species; *(iv)* insect life cycle stage; (*v) Salmonella* serotype; (*vi)* load per contaminated subject; *(vii)* duration of exposure; *(viii)* declaration of surface disinfection of the insect; (*ix)* persistence in insect (days); *(x)* author. The data synthesis also differentiated the complete metamorphosis insects (holometabolous) and the incomplete metamorphosis insects (heterometabolous), since the complete metamorphosis insects may face an extensive change in microbiota between the larval and adult stages due to the gut replacement during pupation^48,49^, unlike incomplete metamorphosis insects. For each category of metamorphosis, we divided the persistence data for two conditions: (*i)* persistence of *Salmonella* in insects subjected to an exposure event following a period of non-exposure (hereinafter referred to as single exposure); (*ii)* persistence of *Salmonella* in insects continuously exposed to contaminated substrate.

The collected data were synthesized and visually displayed in figures reporting for each insect species the longest persistence of *Salmonella* in insect and/or feces. In addition, some studies reported the variation of *Salmonella* counts during the experiment; such data were displayed in graphs created for each insect species reporting the variation of *Salmonella* counts during its persistence in insects or feces. When *Salmonella* counts were not reported in a specific time frame, the values were manually added for technical reasons without affecting the persistence curve trends.

### Quality assessment

Quality assessment was carried out considering relevant aspects for the design of an experimental contamination study: (*i)* use of non-contaminated control groups kept under the same experimental conditions; *(ii)* verification of absence of the target microorganism in individuals to be experimentally contaminated; (*iii)* description of the *Salmonella* serotype used for the experimental contamination; *(iv)* use of standardized analytical methods for detection and/or quantification of the target microorganism; *(v)* characteristics of farming method similar to industrial farming.

Quality assessment of included studies was carried out by one reviewer (S.B.) and verified by a second reviewer (J.P.F.). For each of the five questions in the quality assessment, a positive answer instigated the assignment of one point, while a negative answer resulted in the attribution of 0 points so that at the end of the assessment, a score was obtained for each study with a maximum of 5 and a minimum of 0.

### Reporting summary

Further information on research design is available in the Nature Research Reporting Summary linked to this article.

### Supplementary information

Supplementary Material

Reporting summary

## Data Availability

The authors declare that all data supporting the findings of this study are available in the paper and in supplementary information.
