# Supplementary material for: A systematic review of experimental studies on Salmonella persistence in insects
Source: NPJ Sci Food. 2023 Aug 28;7:44. doi: 10.1038/s41538-023-00223-0 (PMC10462725; doi:10.1038/s41538-023-00223-0)
Supplement: Supplementary file 1 — Supplementary Material [file 41538_2023_223_MOESM1_ESM.pdf]

Supplementary Table 1

Keywords and search strategy used for each investigated scientific literature database.

EMBASE	('acridia':ti,ab,kw OR 'cricket':ti,ab,kw OR 'acheta':ti,ab,kw OR 'gryllus':ti,ab,kw OR 'gryllodes':ti,ab,kw OR 'hieroglyphus':ti,ab,kw OR 'melanoplus':ti,ab,kw OR 'oxya':ti,ab,kw OR 'black soldier':ti,ab,kw OR 'hermetia':ti,ab,kw OR 'blow fly':ti,ab,kw OR 'chrysomya':ti,ab,kw OR 'coleoptera':ti,ab,kw OR 'beetle*':ti,ab,kw OR 'Tribolium':ti,ab,kw OR 'housefly':ti,ab,kw OR 'musca':ti,ab,kw OR 'diptera':ti,ab,kw OR 'grasshopper':ti,ab,kw OR 'orthoptera':ti,ab,kw OR 'Schistocerca':ti,ab,kw OR 'insect*':ti,ab,kw OR 'lepidoptera':ti,ab,kw OR 'locust':ti,ab,kw OR 'locusta':ti,ab,kw OR 'mealworm':ti,ab,kw OR 'alphitobius':ti,ab,kw OR 'tenebrio':ti,ab,kw OR 'palm weevil':ti,ab,kw OR 'sago':ti,ab,kw OR 'rhynchophorus':ti,ab,kw OR 'silkworm':ti,ab,kw OR 'bombyx':ti,ab,kw OR 'superworm':ti,ab,kw OR 'zophobas morio':ti,ab,kw OR 'insect'/exp)<br>AND<br>('salmonell*':ti,ab,kw OR 'salmonella'/exp)<br>AND<br>('expos*':ti,ab,kw OR 'inoculat*':ti,ab,kw OR 'persisten*':ti,ab,kw OR 'dynamic*':ti,ab,kw OR 'reduc*':ti,ab,kw OR 'resistan*':ti,ab,kw OR 'stability':ti,ab,kw OR 'surviv*':ti,ab,kw OR 'viabil*':ti,ab,kw OR 'endur*':ti,ab,kw OR 'exposure'/exp OR 'inoculation'/exp OR 'persistence'/exp OR 'dynamics'/exp OR 'reduction kinetics'/exp OR 'resistance'/exp OR 'stability'/exp OR 'survival'/exp OR 'viability'/exp OR 'endurance'/exp)<br>AND<br>([embase]/lim OR [preprint]/lim)
Food Science and Technology Abstracts (FSTA)	("Acridia" OR "Cricket" OR "Acheta" OR "Gryllus" OR "Gryllodes" OR "Hieroglyphus" OR "Melanoplus" OR "Oxya" OR "black soldier" OR "Hermetia" OR "blow fly" OR "Chrysomya" OR "beetle*" OR "coleoptera" OR "Tribolium" "housefly" OR "Musca" OR "diptera" OR "grasshopper" OR "Orthoptera" OR "Schistocerca" OR "insect*" OR "lepidoptera" OR "locust" OR "Locusta" OR "mealworm" OR "Alphitobius" OR "Tenebrio" OR "palm weevil" OR "sago" OR "Rhynchophorus" OR "silkworm" OR "Bombyx" OR "superworm" OR "Zophobas")<br>AND<br>("Salmonell*")<br>AND<br>("Expos*" OR "Inoculat*" OR "persisten*" OR "dynamic*" OR "reduc*" OR "resistan*" OR "stability" OR "surviv*" OR "viabil*" OR "endur*")
PUBMED	("Cricket"[Title/Abstract] OR "Acheta"[Title/Abstract] OR "Gryllus"[Title/Abstract] OR "Gryllodes"[Title/Abstract] OR "Hieroglyphus"[Title/Abstract] OR "Melanoplus"[Title/Abstract] OR "Oxya"[Title/Abstract] OR "black soldier"[Title/Abstract] OR "Hermetia"[Title/Abstract] OR "blow fly"[Title/Abstract] OR "Chrysomya"[Title/Abstract] OR "coleoptera"[Title/Abstract] OR "beetle*"[Title/Abstract] OR "Tribolium"[Title/Abstract] OR "housefly"[Title/Abstract] OR "Musca"[Title/Abstract] OR "diptera"[Title/Abstract] OR "grasshopper"[Title/Abstract] OR "Orthoptera"[Title/Abstract] OR "insect*"[Title/Abstract] OR "lepidoptera"[Title/Abstract] OR "locust"[Title/Abstract] OR "Locusta"[Title/Abstract] OR "Schistocerca"[Title/Abstract] OR

	"mealworm"[Title/Abstract] OR "Alphitobius"[Title/Abstract] OR "Tenebrio"[Title/Abstract] OR "palm weevil"[Title/Abstract] OR "sago"[Title/Abstract] OR "Rhynchophorus"[Title/Abstract] OR "silkworm"[Title/Abstract] OR "Bombyx"[Title/Abstract] OR "superworm"[Title/Abstract] OR "Zophobas"[Title/Abstract] OR "insecta"[MeSH Terms])<br>AND<br>("Salmonell*"[Title/Abstract] OR "Salmonella"[MeSH Terms])<br>AND<br>("survival"[MeSH Terms] OR "Expos*"[Title/Abstract] OR "Inoculat*"[Title/Abstract] OR "persisten*"[Title/Abstract] OR "dynamic*"[Title/Abstract] OR "reduc*"[Title/Abstract] OR "resistan*"[Title/Abstract] OR "endur*"[Title/Abstract] OR "stability"[Title/Abstract] OR "surviv*"[Title/Abstract] OR "viabil*"[Title/Abstract])
WOS Core Collection	TS=(("Acridia" OR "Cricket" OR "Acheta" OR "Gryllus" OR "Gryllodes" OR "Hieroglyphus" OR "Melanoplus" OR "Oxya" OR "black soldier" OR "Hermetia" OR "blow fly" OR "Chrysomya" OR "coleoptera" OR "beetle*" OR "Tribolium" OR "housefly" OR "Musca" OR "diptera" OR "grasshopper" OR "Orthoptera" OR "Schistocerca" OR "insect*" OR "lepidoptera" OR "locust" OR "Locusta" OR "mealworm" OR "Alphitobius" OR "Tenebrio" OR "palm weevil" OR "sago" OR "Rhynchophorus" OR "silkworm" OR "Bombyx" OR "superworm" OR "Zophobas")<br>AND<br>("Salmonell*")<br>AND<br>("Expos*" OR "Inoculat*" OR "persisten*" OR "dynamic*" OR "reduc*" OR "resistan*" OR "stability" OR "surviv*" OR "viabil*" OR "endur*"))
